# The impact of Zn-doped synthetic polymer materials on bone regeneration: a systematic review

**DOI:** 10.1186/s13287-021-02195-y

**Published:** 2021-02-12

**Authors:** Siyi Wang, Rong Li, Dandan Xia, Xiao Zhao, Yuan Zhu, Ranli Gu, Jungmin Yoon, Yunsong Liu

**Affiliations:** 1 Department of Prosthodontics, Peking University School and Hospital of Stomatology, Beijing, 100081 China; 2 Center of Digital Dentistry, Peking University School and Hospital of Stomatology, Beijing, 100081 China; 3 National Engineering Laboratory for Digital and Material Technology of Stomatology, National Clinical Research Center for Oral Diseases, Beijing Key Laboratory of Digital Stomatology, Beijing, 100081 China; 4 Department of Materials Science and Engineering, College of Engineering, Peking University, Beijing, 100871 China

**Keywords:** Zinc, Synthetic polymers, Bone regeneration, Stem cells

## Abstract

**Introduction:**

To repair bone defects, a variety of bone substitution materials have been used, such as ceramics, metals, natural and synthetic polymers, and combinations thereof. In recent decades, a wide range of synthetic polymers have been used for bone regeneration. These polymers have the advantages of biocompatibility, biodegradability, good mechanical properties, low toxicity, and ease of processing. However, when used alone, they are unable to achieve ideal bone formation. Incorporating zinc (Zn) into synthetic polymers has been considered, as previous studies have shown that Zn^2+^ promotes stem cell osteogenesis and mineral deposition. The purpose of this systematic review was to provide an overview of the application and effectiveness of Zn in synthetic polymers for bone regeneration, whether used alone or in combination with other biomaterials. This study was performed according to the PRISMA guidelines.

**Materials and methods:**

A search of the PubMed, Embase, and the Cochrane Library databases for articles published up to June 2020 revealed 153 relevant studies. After screening the titles, abstracts, and full texts, 13 articles were included in the review; 9 of these were in vitro, 3 were in vivo, and 1 included both in vitro and in vivo experiments.

**Results:**

At low concentrations, Zn^2+^ promoted cell proliferation and osteogenic differentiation, while high-dose Zn^2+^ resulted in cytotoxicity and inhibition of osteogenic differentiation. Additionally, one study showed that Zn^2+^ reduced apatite formation in simulated body fluid. In all of the in vivo experiments, Zn-containing materials enhanced bone formation.

**Conclusions:**

At appropriate concentrations, Zn-doped synthetic polymer materials are better able to promote bone regeneration than materials without Zn.

**Supplementary Information:**

The online version contains supplementary material available at 10.1186/s13287-021-02195-y.

## Background

Bone defects caused by various conditions, such as congenital malformation, trauma, tumor resection, and infection, not only cause great pain but also place tremendous pressure on healthcare systems [1]. Bone tissue engineering (BTE) emerging in recent decades now provides an option for the therapy of bone defects. The method involves combining biomaterials with donor cells to promote bone regeneration, and a variety of biomaterials have been used in BTE to repair and replace traumatized/damaged bone tissue, such as ceramics, metals, natural and synthetic polymers, and combinations thereof [2, 3].

As a commonly used material both in conventional processing methods, like freeze-drying and solvent-casting, and emerging three-dimensional (3D) printing technology, a wide range of synthetic polymers have been applied for bone regeneration, including polylactic acid (PLA), polycaprolactone (PCL), poly (glycolic acid) (PGA), and their copolymers, such as poly (lactic-co-glycolic) acid (PLGA). Approved by the Food and Drug Administration, these polymers are considered biocompatible with natural tissues [4] and have several other advantages. For example, as synthetic materials, their composition, architecture, and physical properties are adjustable; also, these materials are highly reproducible. Moreover, these polymers are degradable, so they can provide mechanical support initially, before gradually degrading to make room for newly formed tissue [5, 6]. However, although synthetic polymers can be osteoconductive, they usually do not achieve the desired effect of complete bone regeneration when used alone [4].

Zinc (Zn), an essential trace element, is usually found in skeletal tissue, and approximately 30% of Zn in the body is contained in bone tissue. Zn plays a significant role in the formation, development, mineralization, and maintenance of healthy bones [7]. Also, as a mediator of bone development and growth, Zn deficiency in humans can lead to diseases such as dwarfism, osteoporosis, and stunted bone development. Previous in vitro studies have demonstrated that Zn^2+^ improves stem cell osteogenesis and enhances mineral deposition [8].

Thus, Zn incorporation is expected to improve the osteogenic ability of synthetic polymer materials, as the release of Zn^2+^ may enhance the osteogenic differentiation of cells for accelerated bone regeneration [9].

However, there is no consensus on the precise role of Zn in these effects, and different types of cells seem to respond differently depending on the Zn^2+^ concentration. In a study by Bertels et al., 0.04–0.08 mM of zinc sulfate (ZnSO_4_) enhanced mineral nodule formation in swine adipose-derived stem cells (swine ASC) in osteogenic media; moreover, the response was biphasic: concentrations of ZnSO_4_ above 0.08 mM were detrimental to cell growth [10]. And Tiffany et al. found that Zn^2+^ in amounts below 0.04 mM still enhanced the cell number and metabolic activity of porcine adipose-derived stem cells (pASCs) [11]. In another study, Xiong et al. showed that 10.91–27.15 μM of Zn^2+^ in cell culture medium significantly enhanced the proliferation and alkaline phosphatase (ALP) activity of mouse bone marrow-derived mesenchymal stem cells (mBMSCs). Similarly, high concentrations (128.58 μM) of Zn have been shown to significantly inhibit ALP activity [12].

In this systematic review, we describe the incorporation of Zn into synthetic polymer materials (whether used alone or in combination with other biomaterials), evaluate the effectiveness of these Zn-doped materials for promoting osteogenesis, and discuss the viable concentration of Zn^2+^ for osteogenesis in different types of cells.

## Methods

### Objectives

The objective of this research was to review the literature on Zn-doped synthetic polymer materials, to provide an overview of the application and effectiveness of incorporating Zn into synthetic polymers to improve their osteogenic ability.

### Guidelines

The PRISMA guidelines were followed in this research [13].

### PICO

P: Synthetic polymer-based materials

I: Incorporation of Zn into synthetic polymers

C: Comparison between synthetic polymers used alone and in combination with Zn

O: Overview of the bone regeneration effect

### Inclusion and exclusion criteria

The following are the inclusion criteria:

- In vitro studies
- In vivo studies with animal bone defect models
- Zn-doped synthetic polymer materials used alone or in combination with other biomaterials
- Materials used in the form of membranes, scaffolds, disks, etc.
- Published in English

The following are the exclusion criteria:

- Synthetic polymer materials not used
- No control group

### Search strategy

The PubMed, Embase, and Cochrane Library databases were searched for studies about the application and effectiveness of Zn-doped synthetic polymer materials for promoting osteogenic ability, published at any time up to June 2020. The following keywords were used: “Zinc,” “Polymers,” and “Osteogenesis.” Details of the search strategies are provided in Supplementary Materials Tables S1, S2, and S3.

### Study selection and data collection

**Fig. 1:**
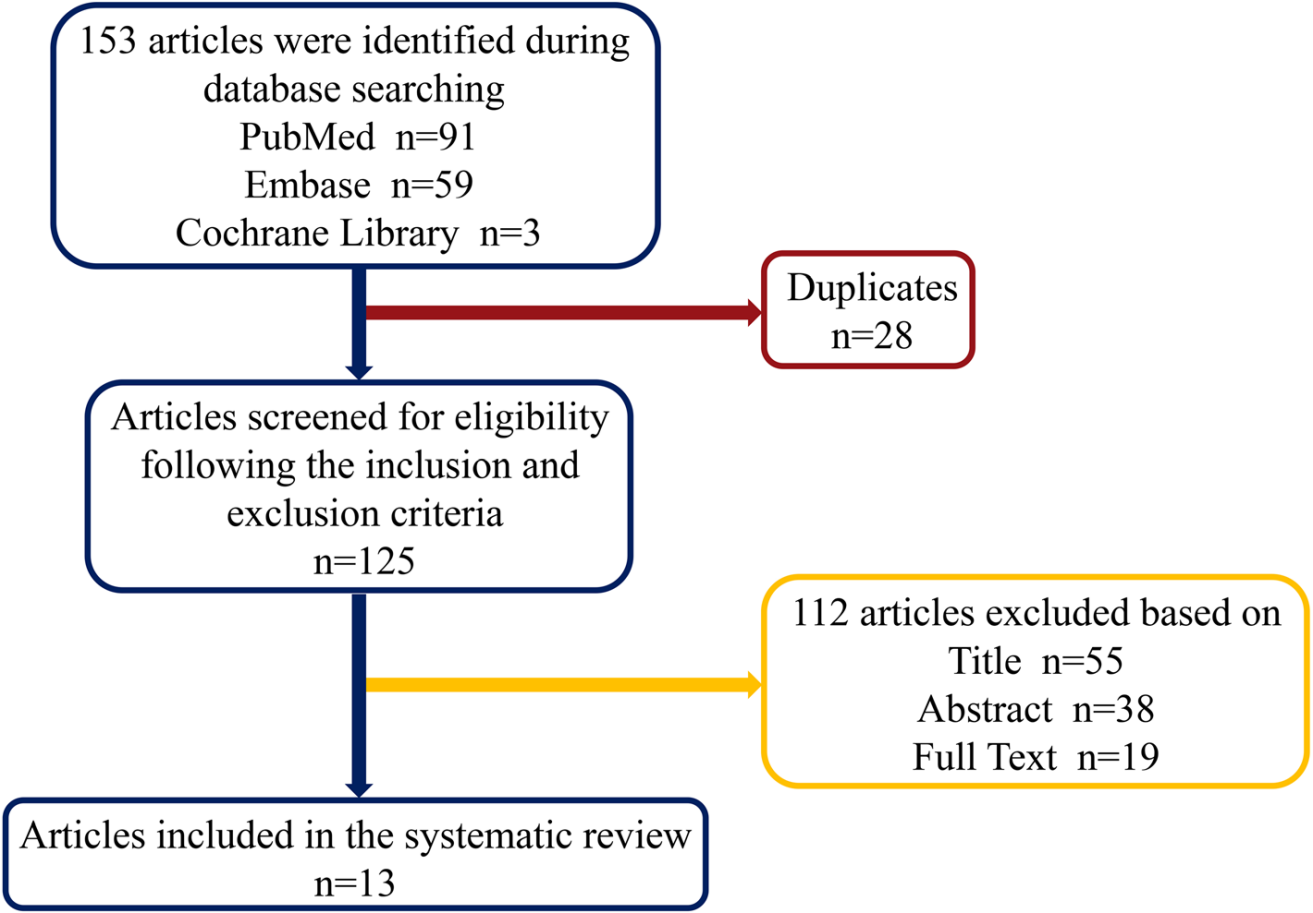
Flow chart of the study selection process

Studies were selected by two reviewers (Li R, Zhao X), who independently screened the titles, abstracts, and full texts of all retrieved articles. Disagreements were resolved by discussion or through consultation with a third reviewer (Wang S). Potentially suitable articles were then assessed according to the inclusion and exclusion criteria, and data were then extracted. A flow chart of the study selection process is shown in Fig. 1.

### Quality assessment

Two other researchers (Zhu Y, Gu R) used the Methodological Index for Non-Randomized Studies (MINORS) scale to evaluate the risk of bias [14, 15]. The revised and validated version of MINORS contains 12 items. Each item is scored from 0 to 2 (0, not reported; 1, inadequately reported; 2, sufficiently reported). The ideal total score is 16 for non-comparative studies and 24 for comparative studies. Disagreements regarding scores were resolved by discussion.

## Results

### Study selection and characteristics

Of the 153 articles screened, 13 were deemed eligible for the review. In total, 28 articles were excluded due to being duplicates, and a further 55, 38, and 19 studies were excluded after screening the title, abstract, and full text, respectively. The contents of the 13 included articles are systematically summarized in Tables 1 and 2, and the results of the quality assessments are listed in Table 3. All included studies had scores above 16 on the MINORS scale, indicating a low risk of bias. In these studies, synthetic polymers were typically used in conjunction with other biomaterials.

**Table 1:** Results of in vitro studies

Author/year	Control group	Experimental group	Zn content	Cell type	Cell proliferation and viability assessment	Cell osteogenic differentiation assessment	Results
Oh et al. 2012 [16]	PLDLA membrane	a. PLDLA membrane combined with bioactive glasses (BG, 70SiO_2_-30CaO), fixed at ~ 7/3 by weight b. PLDLA membrane combined with ZnBG (70SiO_2_-25CaO-5ZnO), fixed at ~ 7/3 by weight	5.41 wt% in ZnBG; 1.62 wt% in the PLDLA-ZnBG membrane	rBMSCs	MTS assay	ALP activity/immunofluorescence staining for BSP/cellular mineralization	No statistical significance was noted in the cell viability assay (*p* > 0.05); the PLDLA-ZnBG group showed the best results in promoting cell osteogenic differentiation and cellular mineralization
Amiri et al. 2016 [17]	Tissue culture polystyrene (TCPs)	a. PES-PEG scaffold, fixed at 7/3 ratio by weight b. Zn_2_SiO_4_-PES-PEG scaffold (PES-PEG scaffold immersed in the ethanol solution containing 1 wt% Zn_2_SiO_4_)	0.59 wt% in the coating	hMSCs	MTT assay	ALP activity/RT-PCR assay/immunofluorescence staining for osteocalcin and osteopontin/calcium content assay/Alizarin Red S (ARS) staining	Higher values of hMSC proliferation rate for Zn_2_SiO_4_-PES-PEG compared to PES-PEG fibrous scaffolds and TCPs; Zn_2_SiO_4_-PES-PEG scaffolds showed the best results in promoting cell osteogenic differentiation and calcium deposition
Bejarano et al. 2016 [18]	Neat PDLLA scaffold	a. PLA/10-BG (60SiO_2_-25CaO-11Na_2_O-4P_2_O_5_; 10 wt% of BG) scaffold b. PLA/30-BG (30 wt% of BG) scaffold c. PLA/10-1CuBG (BG doped with 1 mol% of CuO) scaffold d. PLA/30-1CuBG scaffold e. PLA/10-1ZnBG (BG doped with 1 mol% of ZnO) scaffold f. PLA/30-1ZnBG scaffold g. PLA/10-1Cu1ZnBG (BG doped with 1 mol% of CuO and 1 mol% of ZnO) scaffold h. PLA/30-1Cu1ZnBG scaffold	0.11 wt% in PLA/10-1ZnBG and PLA/10-1Cu1ZnBG; 0.32 wt% in PLA/30-1ZnBG and PLA/30-1Cu1ZnBG;	ST-2 cells	CCK-8 assay	ALP activity	Neat PDLLA scaffolds and scaffolds with 10 wt% of BG showed high cell viability, and the scaffolds with 30 wt% of the zinc-doped BG did not generate significant cytotoxicity; compared to other groups, the PLA/30-1ZnBG scaffold showed the highest ALP activity values
Deng et al. 2018 [19]	Porous sulfonated PEEK (SPEEK) disk	a. Ag-SPEEK disk (SPEEK disk immersed in Ag^+^ solution) b. Zn-SPEEK disk (SPEEK disk immersed in Zn^2+^ solution) c. Ag/Zn-SPEEK disk	18.51 wt% in the coating	Human osteoblast-like MG-63 cells	CCK-8 assay	ALP activity/RT-PCR assay	Zn-containing SPEEK significantly promoted cell proliferation in the initial phase at low zinc concentration; the Ag/Zn-SPEEK surface had the best effect of promoting differentiation of MG-63 cells
Rajzer et al. 2019 [20]	Pure PCL membrane	a. PCL-A2 (BG: 40SiO_2_-54CaO-6P_2_O_5_, 4 wt%) membrane b. PCL-A2Zn5 (BG: 49CaO-5ZnO-6P_2_O_5_-40SiO_2_, 4 wt%) membrane	5.08 wt% in A2Zn5; 0.2 wt% in PCL-A2Zn5	Human osteoblasts cells		ALP activity/bioactivity was evaluated by examining the formation of apatite layer in simulated body fluid (SBF)	BG doped with Zn favors ALP expression in comparison with pure PCL membrane; the surface of PCL-A2 membranes showed the most apatite formation in SBF
Telgerd et al. 2019 [21]	Tissue culture plate (TCP)	a. PLLA nanofiber scaffold b. Zn-Cu-imidazole metal-organic framework (MOF)-coated PLLA scaffold (PLLA@MOF)	9.1 wt% in the coating	Human adipose tissue-derived mesenchymal stem cells	MTT assay	ALP activity/calcium content assay	PLLA@MOF showed good biocompatibility and provided favorable adhesion and proliferation of cells; PLLA@MOF showed the highest ALP activity and calcium deposition
Li et al. 2019 [22]	Poly (amino acids) scaffold (PAA)	a. PAA-0.025M scaffold (PAA powder dispersed into 0.025 mol/L zinc chloride solutions) b. PAA-0.05M scaffold (PAA powder dispersed into 0.05 mol/L zinc chloride solutions) c. PAA-0.1M scaffold (PAA powder dispersed into 0.1 mol/L zinc chloride solutions)	No specific content in the final scaffolds	BMSCs	CCK-8 assay	ALP activity/Alizarin Red S staining	PAA-0.025M and PAA-0.05M promoted cell proliferation, while PAA-0.1M exhibited cytotoxicity; the highest ALP activity and calcium nodules were found with PAA-0.05M
Neto et al. 2019 [23]	Biphasic calcium phosphate (BCP) scaffold coated with PCL/PDLA/PEA/PEU	a. BCP-6Sr scaffold (BCP doped with 6 mol% Sr^2+^) b. BCP-6Sr2Mg scaffold (BCP-6Sr doped with 2 mol% Mg^2+^) c. BCP-6Sr2Zn scaffold (BCP-6Sr doped with 2 mol% Zn^2+^) d. BCP-6Sr2Mg2Zn scaffold (BCP-6Sr doped with 2 mol% Mg^2+^ and 2 mol% Zn^2+^) *All the scaffolds were coated with PCL/PDLA/PEA/PEU	0.8 mol% in BCP-6Sr2Zn scaffold; 0.68 mol% in BCP-6Sr2Mg2Zn scaffold			Biomineralization capacity was analyzed by immersing the scaffolds in SBF	All the composite scaffolds exhibited calcium phosphate microspheres deposition in SBF
Liang et al. 2020 [24]	PLGA/CPC scaffold, fixed at 3/17 by weight	a. PLGA/CPC-Si scaffold (2.6 wt% of CaSiO_3_) b. PLGA/CPC-Zn scaffold (15 wt% of Zn-TCP) c. PLGA/CPC-Si/Zn scaffold (5 wt% of Zn_2_SiO_4_)	2.6 wt% in PLGA/CPC-Zn scaffold; 2.9 wt% in PLGA/CPC-Si/Zn scaffold	rBMSCs		RT-PCR assay/immunofluorescence staining for BMP-2	rBMSCs on the PLGA/CPC-Si/Zn scaffold showed the highest osteogenic differentiation effect
Kandasamy et al. 2020 [25]	PCP: CMC/PVP scaffold	a. PC: (Zn-Mn HAP) scaffold (Zn = Mn = 0.05 M) b. PC1: (Zn-Mn HAP) scaffold (Zn = Mn = 0.1 M) c. PC 20: PC/CMC/PVP scaffold (20 wt% of PC) d. PC 40: PC/CMC/PVP scaffold (40 wt% of PC) e. PC 60: PC/CMC/PVP scaffold (60 wt% of PC) f. PC1–20: PC1/CMC/PVP scaffold (20 wt% of PC1) g. PC1–40: PC1/CMC/PVP scaffold (40 wt% of PC1) h. PC1–60: PC1/CMC/PVP scaffold (60 wt% of PC1)	No specific content in the final scaffolds	Human osteoblast cells (HOS)	MTT assay	Formation of minerals as crystals was analyzed by immersing the scaffolds in SBF	PC1–60 fiber had the highest cell proliferation and attachment values; PC1–60 were selected to perform the biomineralization activity in SBF solution; with increased soaking time, the apatite formation on the sample surface increased

*PLDLA* poly-l-d,l-lactic acid, *MTS* 3-(4,5-dimethylthiazol-2-yl)-5(3-carboxymethonyphenol)-2-(4-sulfophenyl)-2H-tetrazolium, *rBMSCs* rat bone marrow mesenchymal stem cells, *PES* polyethersulphone, *PEG* polyethyleneglycol, *MTT* 3-(4,5-dimethylthiazolyl-2)-2,5-diphenyltetrazolium bromide, *hMSCs* human mesenchymal stem cells, *PDLLA* poly (d, l-lactide), *CCK-8* Cell Counting Kit-8, *ST-2* bone marrow stromal cells, *PEEK* polyetheretherketone, *OD* optical density, *PCL* polycaprolactone, *PLLA* poly-l-lactic acid, *PAA* poly (amino acids), *PDLA* poly (dl-lactide), *PEA* poly (ester amide), *PEU* poly (ester urea), *PLGA* poly (lactic-co-glycolic acid), *CPC* calcium phosphate cement, *CMC* carboxymethyl cellulose, *PVP* polyvinyl pyrrolidone, *HAP* hydroxyapatite, *Runx2* runt-related transcription factor 2, *Col I* collagen type 1, *ALP* alkaline phosphatase, *OCN* osteocalcin, *BMP-2* bone morphogenetic protein-2

**Table 2:** Results of in vivo studies

Author/year	Control group	Experimental group	Zn content	Animal model/bone defect preparation	Bone regeneration measurement	Results
Ahmadzadeh et al. 2016 [26]	Control HA	Carbonate hydroxyapatite (cHA) and Zn-Mg-HA nanoparticles (mixed in 1:1 wt% ratio) combined with polyvinyl alcohol (PVA) hydrogel to form a composite graft (CZM-HA graft)	7.85 wt% in Zn-Mg-HA; 1.96 wt% in the CZM-HA graft	Tibia of male New Zealand albino rabbits; right distal tibia; two holes (4 mm diameter, 2 mm depth); two disk-shaped bone grafts (CZM-HA and control) were embedded.	Micro-CT evaluation/bone quantification with the ImageJ software/SEM-EDX analyses/H&E staining	A few Haversian canals were observed in the CZM-HA graft section; only red blood cells (RBC) and immature bone tissue were seen in control graft section.
Toledano et al. 2020 [27]	SiO_2_-NP-doped membrane (HOOC-Si-membrane, the membrane was polymer blend [(MMA)_1_-co-(HEMA)_1_/(MA)_3_-co-(HEA)_2_], comprising 5 wt% of SiO_2_ nanoparticles)	a. SiO_2_-NP-doped membrane functionalized with Zn (Zn-HOOC-Si-membrane, 3 μg Zn/mg membrane) b. SiO_2_-NP-doped membrane functionalized with Dox (Dox-HOOC-Si-membrane, 76.2 μg Dox/mg membrane)	0.3 wt% in the Zn-HOOC-Si-membrane	Skull of New Zealand-breed experimentation white rabbits; each side of the skull midline; four bone defects (8 mm diameter, 3 mm depth); randomly allocated membrane of each three groups was used for three bone defects, the fourth was not covered.	Micro-CT evaluation/bone quantification with the ImageJ software/von Kossa silver nitrate stain/toluidine blue staining/fluorescence morphometric studies of the deposition of calcein	Bony bridging processes were observed in the Zn-HOOC-Si-membrane group, while in other groups, the bone only regenerated at the defect edge, without evidence of bridging.
Liang et al. 2020 [24]	PLGA/CPC scaffold, fixed at 3/17 by weight	a. PLGA/CPC-Si scaffold (2.6 wt% of CaSiO_3_) b. PLGA/CPC-Zn scaffold (15 wt% of Zn-TCP) c. PLGA/CPC-Si/Zn scaffold (5 wt% of Zn_2_SiO_4_)	2.6 wt% in PLGA/CPC-Zn scaffold; 2.9 wt% in PLGA/CPC-Si/Zn scaffold	Femur of SD rats; two femurs of each rat; cylindrical defects (2 mm diameter, 5 mm height); different scaffolds were implanted into each side.	Micro-CT evaluation/H&E staining/Masson’s trichrome staining	Compared to other 3 groups, the PLGA/CPC-Si/Zn scaffolds yielded a substantial increase in the amount of regenerated bone volume.
Toledano et al. 2020 [28]	Control nanostructured membranes (Ms, a novel polymer blend polymethylmethacrylate (PMMA))	a. Ms. loaded with calcium (1.5 μg Ca/mg Ms) b. Ms. loaded with zinc (3 μg Zn/mg Ms) c. Ms. loaded with TiO_2_ nanoparticles (6% of TiO_2_ nanoparticles) d. Ms. loaded with human recombinant bone-morphogenetic protein 2 (1.0 μg of protein)	0.3 wt% in Ms. loaded with zinc group	Skull of New Zealand-breed experimentation white rabbits; each side of the skull midline; six bone defects (6 mm diameter, 3 mm depth); randomly assigned membrane was used for five bone defects, the sixth was left uncovered.	Micro-CT evaluation/bone quantification with the ImageJ software/von Kossa silver nitrate stain/toluidine blue staining/fluorescence morphometric studies of the deposition of calcein	Zn-Ms produced the highest amount of new bone among groups and showed a bridge-like network between the areas of the new bone.

*NPs* nanoparticles, *Dox* doxycycline, *PLGA* poly (lactic-co-glycolic acid), *CPC* calcium phosphate cement

**Table 3:** MINORS bias scale

Evaluation	[16]	[17]	[26]	[18]	[19]	[20]	[21]	[22]	[23]	[27]	[24]	[28]	[25]
Clearly stated aim	2	2	2	2	2	2	2	2	2	2	2	2	2
Contemporary groups	2	2	2	2	2	2	2	2	2	2	2	2	2
Prospective collection of data	2	2	2	2	2	2	2	2	2	2	2	2	2
Sample randomization	0	0	2	0	0	0	0	0	0	2	2	2	0
Test group: Zn content in the materials: 0 (not reported), 1 (materials coated with Zn or rough mixing ratio of Zn), 2 (precise mixing ratio of Zn)	2	1	2	2	1	2	1	1	2	2	2	2	1
Measurements standardization	2	2	2	2	2	2	2	2	2	2	2	2	2
Condition of the samples during measurements	2	2	2	2	2	2	2	2	2	2	2	2	2
Measurements method	2	2	2	2	2	2	2	2	2	2	2	2	2
Endpoints appropriate to the aim of the study	2	2	2	2	2	2	2	2	2	2	2	2	2
Unbiased assessment of the study endpoint	0	0	0	0	0	0	0	0	0	0	0	0	0
Baseline equivalence of groups	2	2	2	2	2	2	2	2	2	2	2	2	2
Adequate statistical analyses	2	2	0	2	2	2	2	2	2	2	2	2	0
Total Score	20	19	20	20	19	20	19	19	20	22	22	22	17

Each item is scored 0 (not reported), 1 (inadequately reported), or 2 (sufficiently reported). The global ideal score is 16 for non-comparative studies and 24 for comparative studies

Nine of the studies were conducted in vitro, three were in vivo, and one included both in vitro and in vivo experiments. Three of the studies were performed on New Zealand rabbits (tibia or skull) [26–28], and one was performed on Sprague-Dawley (SD) rats (femur) [24]. The in vitro studies were concerned with cell proliferation and viability, and osteogenic differentiation, assessed using MTS, MTT, and CCK-8 assays, and ALP activity, RT-PCR, immunofluorescence staining, Alizarin Red S (ARS) staining, and calcium content assay, respectively.

### Results of individual studies

To assess the effect of Zn-doped synthetic polymer materials on bone regeneration, proliferation/viability and osteogenic differentiation data were obtained, and in vivo osteogenesis evaluations were performed, as discussed above.

#### Cell proliferation/viability assessments

Seven of the thirteen studies assessed cellular proliferation and viability using the MTS assay (*n* = 1) [16], MTT assay (*n* = 3) [17, 21, 25], or CCK-8 assay (*n* = 3) [18, 19, 22]. The results mostly showed that the Zn-containing materials did not exhibit cytotoxicity. However, Li et al. demonstrated that the response of bone marrow-derived mesenchymal stem cells (BMSCs) to a Zn-containing coating was highly dose-dependent [22]. Specifically, an appropriate dose (polyacrylic acid [PAA] powder dispersed in 0.025 or 0.05 mol/L zinc chloride solution; PAA-0.025 M and PAA-0.05 M, respectively) promoted cell proliferation, whereas a high dose (PAA powder dispersed in 0.1 mol/L zinc chloride solution; PAA-0.1 M) exhibited cytotoxicity [22].

#### Cell osteogenic differentiation assessments

Of the 13 articles, 10 assessed osteogenic differentiation based on ALP activity, RT-PCR, immunofluorescence staining, ARS staining, and calcium assay. One study used all of the abovementioned evaluation methods [17], and the others used at least one of the techniques. Overall, Zn-containing materials significantly promoted osteogenic differentiation compared to the control groups without Zn. Amiri et al. fabricated a Zn_2_SiO_4_-PES-PEG scaffold and showed the osteogenic differentiation rate of human mesenchymal stem cells (hMSCs) on this scaffold was increased compared to that of PES-PEG fibrous scaffolds [17]. Li et al. demonstrated that, consistent with cell proliferation results, the PAA-0.025M and PAA-0.05M groups exhibited higher ALP activity and more calcium nodules compared to the PAA and PAA-0.1M groups [22]. Interestingly, Rajzer et al. found that PCL-A2Zn5 (PCL combined with 49CaO-5ZnO-6P2O5-40SiO2 bioactive glass [BG]) membranes promoted ALP expression, whereas in simulated body fluid (SBF), apatite formation on the surface of the PCL-A2 (PCL combined with 40SiO2-54CaO-6P2O5) membranes was markedly increased compared to pure PCL and PCL-A2Zn5 membranes [20].

It is worth mentioning that some of the studies added other metal ions to the polymer materials besides Zn^2+^ and thus achieved better results with respect to promoting bone regeneration. Deng et al. reported that a silver (Ag)/Zn-codecorated SPEEK surface showed enhanced cell proliferation and osteogenic differentiation compared to a Zn-SPEEK surface [19]. Liang et al. compared the efficacy of PLGA/CPC-Zn scaffolds and PLGA/CPC-Si/Zn scaffolds in terms of osteogenesis; their results showed that the expression of bone morphogenetic protein 2 (BMP-2) was higher in the PLGA/CPC-Si/Zn scaffolds [24].

#### In vivo experiments

**Fig. 2:**
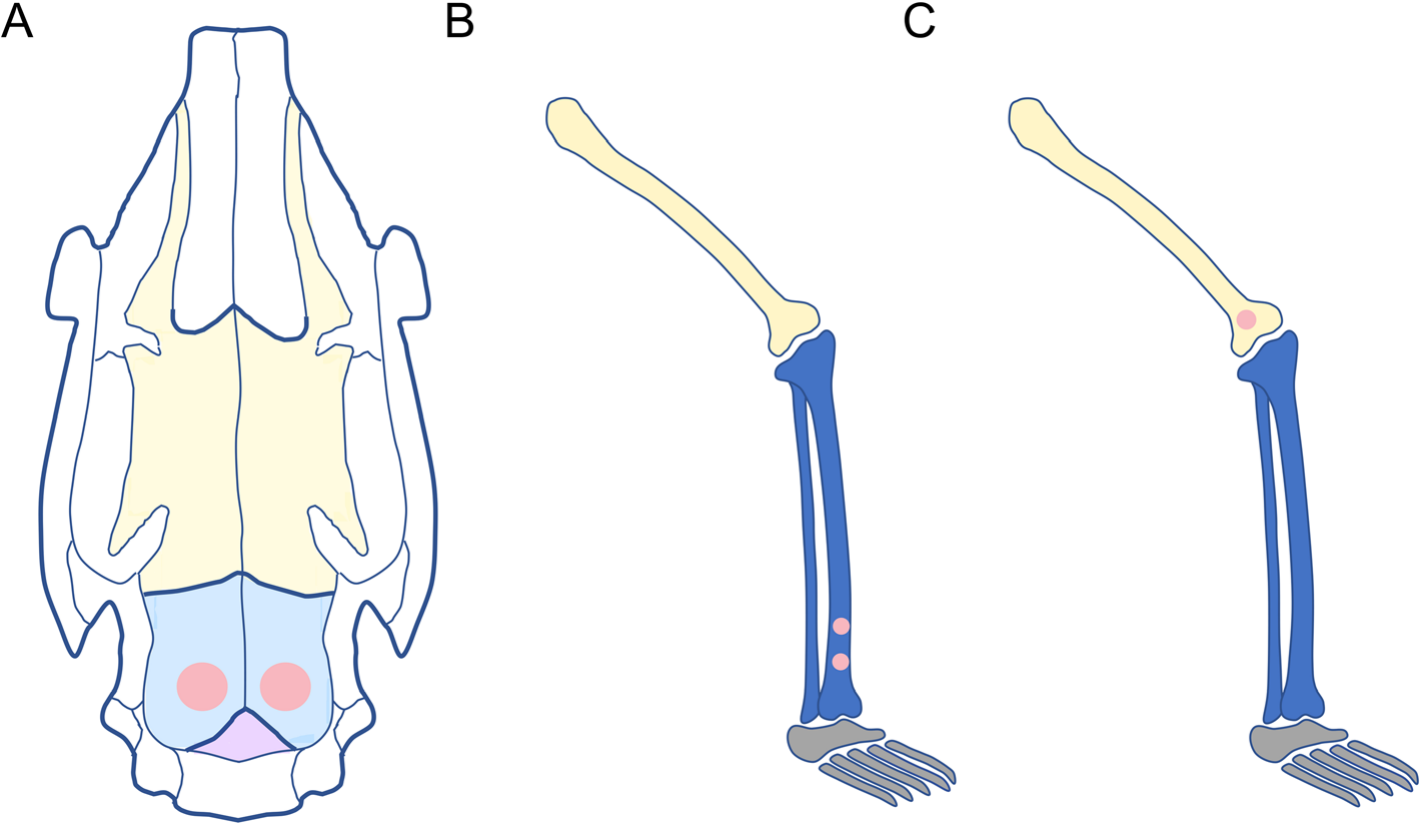
In vivo experiments with animal bone defect models. **a** Skull defects in rabbits [27, 28]. **b** Tibia defects in rabbits [26]. **c** Femur defects in SD rats [24]

In the in vivo experiments, bone defect sites were created in the tibia, femur, and skull, typically in New Zealand rabbits or SD rats (Fig. 2). All of the studies showed that Zn-containing materials can play a role in promoting bone formation, consistent with in vitro studies. It should be noted that Liang et al. demonstrated that PLGA/CPC-Si/Zn scaffolds yielded a substantial increase in the amount of regenerated bone volume over the PLGA/CPC-Zn scaffolds [24].

## Discussion

The unique advantages of synthetic polymers include biocompatibility, biodegradability, good mechanical properties, and low toxicity; additionally, their shape, porosity, and mechanical properties can be well defined and controlled, and they can be stably mass-produced [29]. However, hydrophilia deficiencies and a lack of bioactivity compromise the ability of synthetic polymers to facilitate biomaterial-host interactions. Furthermore, they typically show poor antibacterial properties, and there is some concern that their degradation products generate an acidic environment conducive to inflammation [5]. Therefore, synthetic polymers are commonly combined with other biomaterials to obtain two-dimensional or 3D scaffolds that promote the desired cell behavior and tissue regeneration.

Some of the 13 studies included in our review support the conclusion that the addition of BGs can address the abovementioned issues. In the study of Rajzer et al., an SBF bioactivity test demonstrated that the presence of BG on PCL membranes induced mineralization, thus indicating its bioactivity [20]. As a solution to the problem of acidic products, Oh et al. showed that the ions dissolved from BGs neutralize the acidic environment caused by polymers, and reported significantly higher expression of ALP and osteocalcin in samples containing BG and, especially, ZnBG [16]. To improve antibacterial performance, Bejarano et al. incorporated BG doped with copper (Cu) and Zn into poly (d, l-lactide) (PDLLA). The results showed that the Cu- and Zn-doped BG improved antibacterial activity [18].

When used alone, synthetic polymers usually cannot achieve the desired effect of complete bone regeneration. In recent years, biodegradable metals (BMs) have gradually become a hotspot in the field of biomedical materials used in BTE because of their good biocompatibility, degradability, and appropriate mechanical properties. Among BMs, Zn has received extensive attention due to its satisfying biological properties in promoting bone regeneration [30].

**Fig. 3:**
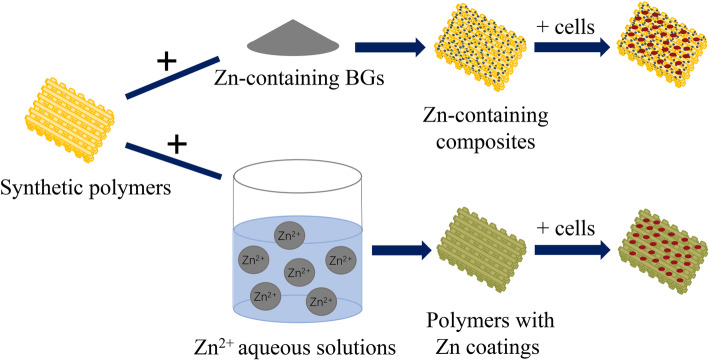
Zn incorporated into polymers as composites or coatings

Thus, incorporating Zn into the synthetic polymers combines the advantages of both and continues to receive much attention. Previous studies have indicated that Zn can regulate cells, which stimulates osteoblastogenesis and attenuates osteoclastogenesis. In the process of bone formation, Zn regulates the secretion and expression of osteogenic markers, such as ALP, and the deposition of minerals [31, 32]. However, the role of Zn in the process of bone regeneration depends on its content. In the selected articles, Zn is usually incorporated into the polymers as a composite or coating (Fig. 3). The content of Zn in the composite materials ranged from 0.11 to 2.9 wt%; the results indicated that, in this range, Zn exerts a satisfactory bone-promoting function. While in the Zn-containing coatings, the concentration of Zn did not show consistency, generally within the range of 20 wt%, as the surface area, thickness, and density of the coatings may also count greatly in the Zn-release process. Nevertheless, it can be concluded that Zn-containing coatings have viable osteogenic effects. Moreover, several studies have indicated that Zn-coating bone implants enhance osseointegration [33, 34].

Previous studies have discussed the possible toxic effect of Zn on cells. Some researchers pointed out that in the case of BGs, ~ 5 mol% Zn doping is the upper limit before cytotoxicity occurs. Additionally, only the highest concentration (5 mg/L) elution extracts inhibited the growth of mouse embryonic fibroblasts (MEFs) [35–37]. However, Neščáková et al. [7] showed that Zn-doped BGs (~ 8 mol% of ZnO) did not exhibit cytotoxicity towards MG-63 or MEF cells; this can be explained by the relatively low Zn^2+^ concentration released into the medium (1.2 mg/L). Thus, it can be inferred that, in terms of the likely effects of Zn on the biological behavior of cells, the release concentration of Zn^2+^ in aqueous solutions is a more accurate indicator than the proportion of Zn in materials.

In aqueous solutions, different cells respond differently to the Zn^2+^ concentration (Table 4).

It has been shown that Zn^2+^ improves the adhesion, spreading, proliferation, and migration of vascular smooth muscle cells, up to concentrations of 60–80 μM (3.9–5.2 mg/L), while higher Zn^2+^ concentrations (80–120 μM, 5.2–7.8 mg/L) cause opposite responses [38]. Aina et al. [34, 35] reported that a Zn^2+^ concentration of 1.1 mg/L increased the proliferation rate of endothelial cells, whereas cytotoxicity was observed at 2.7 mg/L. Yamaguchi et al. [39] demonstrated that, in the presence of Zn at concentrations of 10^−6^–10^−4^ M (65 μg/L–6.5 mg/L), MC3T3-E1 osteoblasts showed an increase in osteoprotegerin, Runx-2, and regucalcin mRNA expression. The results presented by the same author indicated that a Zn^2+^ concentration between 10 and 250 μM (0.65 and 16.25 mg/L) suppressed osteoclastogenesis of RAW264.7 [40]. The behavior of human osteoblast-like cell line SaOS-2 was also Zn-concentration-dependent; ALP expression and mineral deposition were stimulated under Zn^2+^ concentrations of 1–10 μM (65 μg/L–0.65 mg/L) but were inhibited at concentrations exceeding 25 μM (1.6 mg/L) [41]. Holloway et al. found that Zn concentrations lower than 10^−4^ mol/L (6.5 mg/L) did not affect osteoclast activity, whereas higher concentrations promoted the proliferation of tartrate-resistant acid phosphatase (TRAP)-positive cells [42]. Under Zn^2+^ concentrations lower than 10^−9^ M (65 ng/L), osteoblasts and primary murine bone marrow stromal cells showed normal proliferation, while osteogenic and adipogenic differentiation were repressed [43]. Wang et al. reported that, without affecting cell proliferation under conditions of long-term stimulation, Zn^2+^-passivated carbon dots (Zn-CDs) with a Zn^2+^ release concentration of 10^−5^ mol/L (0.65 mg/L) achieved the best osteogenic effect in rat bone marrow-derived mesenchymal stem cells (rBMSCs) [44].

**Table 4:** Summary of the effects of Zn^2+^ content on different cells

Source	Cell types	Promoting Zn^2+^ content	Inhibiting Zn^2+^ content	References
Adipose	Swine ASC	0.04–0.08 mM for mineral nodule formation	Above 0.08 mM for cell growth	[10]
	pASCs	Below 0.04 mM for cell number and metabolic activity	–	[11]
Bone	mBMSCs	10.91–27.15 μM for proliferation and ALP activity	128.58 μM for ALP activity	[12]
	MC3T3-E1 osteoblasts	10^−6^–10^−4^ M for osteoprotegerin, Runx-2, and regucalcin mRNA expression	–	[38, 39]
	Human osteoblast-like cell line SaOS-2	1–10 μM for ALP expression and mineral deposition	Exceeding 25 μM for the same functions	[40, 41]
	Osteoclast	10^−12^–10^−4^ mol/L for cell activity	Higher than 10^−4^ to promote the proliferation of TRAP-positive cells	[41, 42]
	Osteoblasts and primary murine bone marrow stromal cells	Lower than 10^−9^ M for proliferation	Lower than 10^−9^ M for osteogenic and adipogenic differentiation	[42, 43]
	rBMSCs	10^−5^ mol/L to achieve the best osteogenic effect	–	[43, 44]
Vascular	Endothelial cells	1.1 mg/L for proliferation	2.7 mg/L to cause cytotoxicity	[34, 35]
	Vascular smooth muscle cells	60–80 μM for cell adhesion, spreading, proliferation, and migration	80–120 μM for the same cellular functions	[37, 38]
Abdomen	RAW264.7	10–250 μM to suppress osteoclastogenesis	–	[39, 40]

**Fig. 4:**
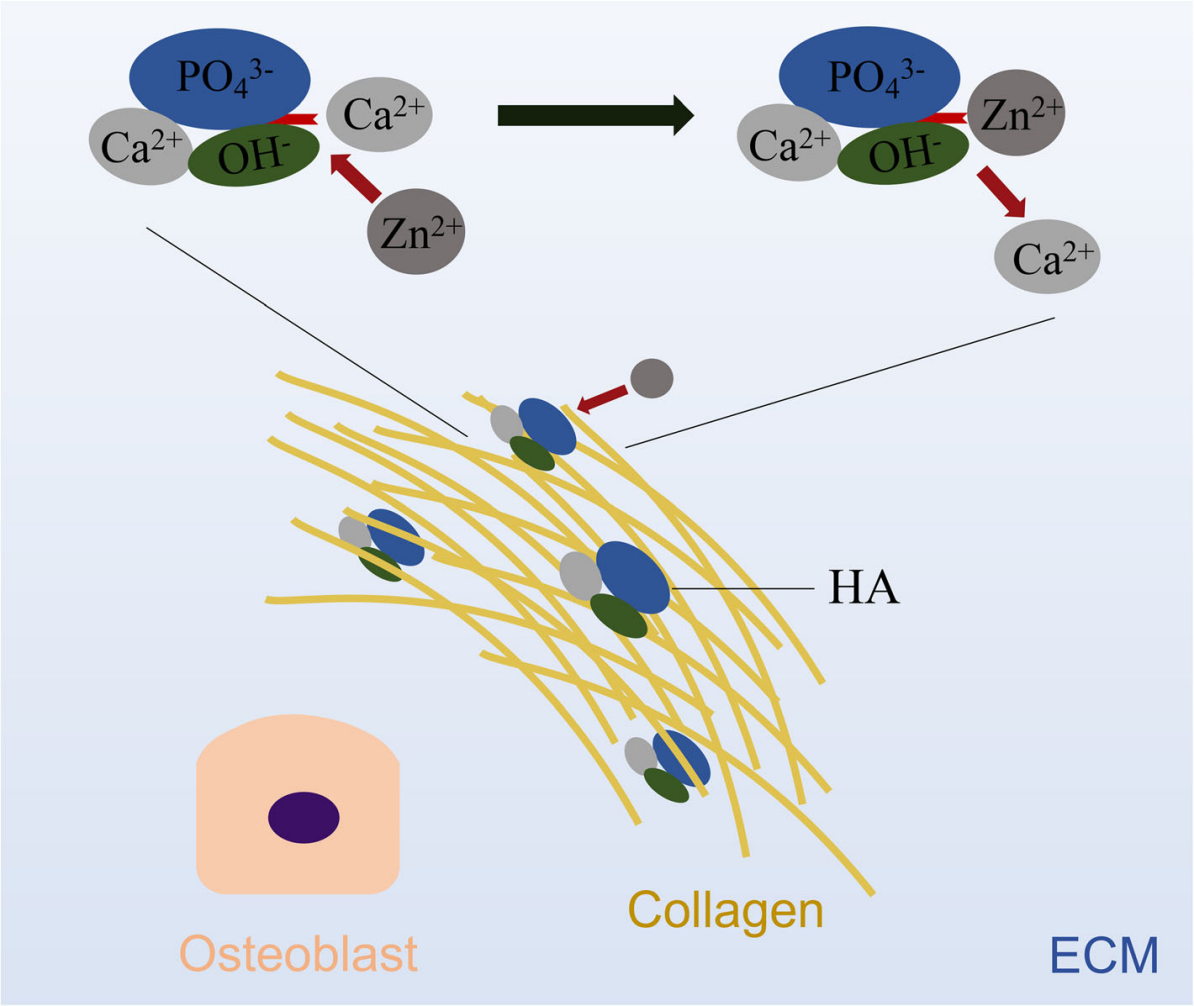
Zn^2+^ prevents HA nucleation by binding to its active growth sites

Rajzer et al. found out that in SBF, BG containing Zn showed less apatite formation on the surface compared to BG without Zn [20]. This could be explained that in SBF, Zn may influence the kinetics of hydroxyapatite (HA) formation and retard HA nucleation [45]. By binding to the active growth sites of HA, Zn^2+^ prevents its nucleation [46] (Fig. 4). Moreover, as the Zn^2+^ content increases, the deposition rate of HA decreases. Previous studies have pointed out that, at low concentrations, the released Zn^2+^ may stimulate osteoblast differentiation, osteogenic differentiation of mesenchymal stem cells (MSCs), and extracellular matrix (ECM) mineralization in vitro [47–49], whereas at high concentrations, Zn^2+^ reduces ECM mineralization and may cause cytotoxicity [30, 31]. It can be concluded that, with appropriate concentrations and release behavior, the addition of Zn to a bone scaffold is likely to promote bone regeneration.

The mechanism by which Zn promotes bone formation has been partially explored but remains unclear. Zhu et al. used pure Zn disks and human mesenchymal stem cells (hMSCs) to show that intracellular Zn^2+^ triggers Ca^2+^ responses by activating the cyclic adenosine monophosphate protein-kinase A (cAMP-PKA) pathway, followed by the mitogen-activated protein kinase (MAPK) pathway. The Gαq-PLC-AKT pathway is also activated by Zn^2+^; the activity in all of these pathways enhances cell growth and differentiation, ECM mineralization, and differential regulation of genes [50]. Similarly, Park et al. suggested that ZnSO_4_ exerts osteogenic effects on human bone marrow-derived mesenchymal stem cells (hBMSCs) by activating RUNX2 via the cAMP-PKA-CREB pathway [51]. Fathi and Farahzadi demonstrated that, in electromagnetic fields, ZnSO_4_ activated the extracellular signal-regulated protein kinase (ERK) 1/2, PKA, and Wnt/β-catenin signaling pathways to promote the osteogenesis of adipose tissue-derived MSCs [52]. Yamaguchi et al. showed that ZnSO_4_ attenuated the nuclear factor-kappa B (NF-κB) pathway activation induced by tumor necrosis factor-alpha (TNF-α), to promote osteoblast mineralization and suppress osteoclast differentiation, and could also mitigate the transforming growth factor-beta/bone morphogenic protein-2 (TGF-β/BMP-2)-induced inhibitory effect of TNF-α on the activation of Smad [39, 40]. In addition, there have been several studies of Zn-containing composite materials. Vimalraj et al. revealed that Zn-silibinin complexes regulated the miR-590/Smad7 signaling pathway to enhance osteoblast differentiation [53]. Fernandes et al. incorporated Zn and citrate into HA nanoparticles and demonstrated osteogenic differentiation of BMSCs in the absence of osteoinductive factors, with the functioning regulated, at least in part, by activation of the canonical Wnt pathway [54]. Using Zn-modified calcium silicate coatings, Yu et al. showed that the TGF-β/Smad signaling pathway plays a significant role in regulating the osteoblastic differentiation of rat bone marrow-derived pericytes [55]. Despite these findings, the mechanism by which Zn-doped synthetic polymer materials promote osteogenesis has not been fully elucidated.

Some of the 13 studies in this review used multiple ions. Deng et al. demonstrated that Ag nanoparticle-decorated and Ag/ZnO-codecorated SPEEK effectively inhibit the reproduction of Gram-positive and Gram-negative bacteria. Moreover, Ag/ZnO-codecorated SPEEK substrates were better able to enhance the biocompatibility and osteodifferentiation of MG-63 cells, likely due to the combined effect of micro-/nanoscale topological cues and Zn induction [19]. In the study of Telgerd et al., Cu not only acted as an angiogenic agent, but also enhanced angiogenesis in vitro and increased the proliferation rate of endothelial cells [21]. Liang et al. showed that silicon (Si) promotes vascularization; they integrated Si-Zn and PLGA microspheres into CPC scaffolds, in which Si^4+^ was released at a faster rate than Zn^2+^ to initially promote angiogenesis and, later, osteogenesis. This was attributed to the biodegradable PLGA microspheres, which allowed for the successional release of Si and Zn. As the sequential vascularization and osteogenesis corresponded to the natural process of bone defect restoration, the PLGA/CPC-Si/Zn group showed better bone regeneration than the PLGA/CPC-Zn group [24]. Therefore, the synergistic effect of different metal ions on the biological properties of synthetic polymers is a topic worthy of further investigation and could be explored using Zn-doped synthetic polymer materials.

Based on the MINORS, the studies in this review were of satisfactory quality. However, no clinical studies were obtained through our database searches, indicating that synthetic polymers have not yet been applied clinically in humans. Meanwhile, the lack of standardization among the studies prevented us from conducting a meta-analysis.

We believe that the main significance of this systematic review is to prove the addition of Zn to synthetic polymers promotes bone regeneration; moreover, the viable ranges of Zn^2+^ concentrations for different cell types were demonstrated. However, additional research is necessary to uncover the mechanism by which Zn-doped synthetic polymer materials promote bone regeneration and other potential clinical applications. Finally, the effect of Zn addition on other properties (e.g., mechanical, degradation, and biocompatibility properties) should be examined further.

## Conclusion

Synthetic polymers are commonly used in bone regeneration; however, their ability to promote bone formation is limited when used alone. The introduction of Zn may address this issue. In this systematic review, an overview of the application and effectiveness of Zn incorporation into synthetic polymers is presented. The results showed that, with appropriate concentrations and release behavior, Zn-containing synthetic polymers have the potential to promote bone regeneration. However, the mechanism of action and feasibility for clinical application require further study.

## Supplementary Information

**Additional file 1 **: **Table S1.** Search strategies in PubMed database and related results.

**Additional file 2 **: **Table S2.** Search strategies in Embase database and related results.

**Additional file 3 **: **Table S3.** Search strategies in Cochrane Library database and related results.

## Data Availability

All supporting data are included in the article and its supplementary files.

## Abbreviations

Zn: Zinc
3D: Three-dimensional
PLA: Polylactic acid
PCL: Polycaprolactone
PGA: Poly (glycolic acid)
PLGA: Poly (lactic-co-glycolic) acid
ZnSO_4_: Zinc sulfate
swine ASC: Swine adipose-derived stem cells
pASCs: Porcine adipose-derived stem cells
ALP: Alkaline phosphatase
mBMSCs: Mouse bone marrow-derived mesenchymal stem cells
PLDLA: Poly-l-d,l-lactic acid
MTS: 3-(4,5-Dimethylthiazol-2-yl)-5(3-carboxymethonyphenol)-2-(4-sulfophenyl)-2H-tetrazolium
rBMSCs: Rat bone marrow mesenchymal stem cells
PES: Polyethersulphone
PEG: Polyethyleneglycol
MTT: 3-(4,5-Dimethylthiazolyl-2)-2,5-diphenyltetrazolium bromide
hMSCs: Human mesenchymal stem cells
PDLLA: Poly (d, l-lactide)
CCK-8: Cell Counting Kit-8
ST-2: Bone marrow stromal cells
PEEK: Polyetheretherketone
OD: Optical density
PLLA: Poly-l-lactic acid
PAA: Poly (amino acids)
PDLA: Poly (dl-lactide)
PEA: Poly (ester amide)
PEU: Poly (ester urea)
PLGA: Poly (lactic-co-glycolic acid)
CPC: Calcium phosphate cement
CMC: Carboxymethyl cellulose
PVP: Polyvinyl pyrrolidone
HAP: Hydroxyapatite
Runx2: runt-related transcription factor 2
Col I: Collagen type 1
OCN: Osteocalcin
BMP-2: Bone morphogenetic protein-2
NPs: Nanoparticles
Dox: Doxycycline
SD: Sprague-Dawley
ARS: Alizarin Red S
BMSCs: Bone marrow-derived mesenchymal stem cells
PAA: Polyacrylic acid
BG: Bioactive glass
SBF: Simulated body fluid
BMP-2: Bone morphogenetic protein 2
Cu: Copper
MEFs: Mouse embryonic fibroblasts
TRAP: Tartrate-resistant acid phosphatase
HA: Hydroxyapatite
MSCs: Mesenchymal stem cells
ECM: Extracellular matrix
cAMP-PKA: Cyclic adenosine monophosphate protein-kinase A
MAPK: Mitogen-activated protein kinase
ERK: Extracellular signal-regulated protein kinase
NF-κB: Nuclear factor-kappa B
TNF-α: Tumor necrosis factor-alpha
TGF-β: Transforming growth factor-beta
Si: Silicon
