# Supplementary material for: The impact of Zn-doped synthetic polymer materials on bone regeneration: a systematic review
Source: Stem Cell Res Ther. 2021 Feb 12;12:123. doi: 10.1186/s13287-021-02195-y (PMC7881550; doi:10.1186/s13287-021-02195-y)
Supplement: Supplementary file 1 — Additional file 1 : Table S1. Search strategies in PubMed database and related results. [file 13287_2021_2195_MOESM1_ESM.docx]

**Table S1.** Search strategies in PubMed database and related results.

| **Search** | **Search Strategies** | **Number of Publications** |
| --- | --- | --- |
| #1 | ("Zinc"[Mesh]) OR (Zn) | 114710 |
| #2 | ("Osteogenesis"[Mesh]) OR (((((((((((((Bone Formation) OR (Ossification)) OR (Ossifications)) OR (Osteoclastogenesis)) OR (Osteoclastogeneses)) OR (Endochondral Ossification)) OR (Endochondral Ossifications)) OR (Ossification, Endochondral)) OR (Ossifications, Endochondral)) OR (Physiologic Ossification)) OR (Ossification, Physiological)) OR (Physiological Ossification)) OR (Ossification, Physiologic)) | 134004 |
| #3 | (("Polymers"[Mesh]) OR (Polymer)) OR (((((((((((((synthetic polymers) OR (polyester)) OR ((PLA) OR (polylactic acid))) OR ((PGA) OR (Polyglycolic acid))) OR ((PLGA) OR (poly(lactic-co-glycolic acid)))) OR ((PCL) OR (Polycaprolactone))) OR ((PU) OR (polyurethane))) OR ((PEG) OR (Polyethylene glycol))) OR ((PBT) OR (polybutylene terephthalate))) OR ((PAA) OR (Polyacrylic acid))) OR ((PEO) OR (polyethylene oxide))) OR ((PVA) OR (polyvinyl alcohol))) OR ((PDO) OR (polydioxanone))) | 1089127 |
| #4 | ((("Zinc"[Mesh]) OR (Zn)) AND ((("Polymers"[Mesh]) OR (Polymer)) OR (((((((((((((synthetic polymers) OR (polyester)) OR ((PLA) OR (polylactic acid))) OR ((PGA) OR (Polyglycolic acid))) OR ((PLGA) OR (poly(lactic-co-glycolic acid)))) OR ((PCL) OR (Polycaprolactone))) OR ((PU) OR (polyurethane))) OR ((PEG) OR (Polyethylene glycol))) OR ((PBT) OR (polybutylene terephthalate))) OR ((PAA) OR (Polyacrylic acid))) OR ((PEO) OR (polyethylene oxide))) OR ((PVA) OR (polyvinyl alcohol))) OR ((PDO) OR (polydioxanone))))) AND (("Osteogenesis"[Mesh]) OR (((((((((((((Bone Formation) OR (Ossification)) OR (Ossifications)) OR (Osteoclastogenesis)) OR (Osteoclastogeneses)) OR (Endochondral Ossification)) OR (Endochondral Ossifications)) OR (Ossification, Endochondral)) OR (Ossifications, Endochondral)) OR (Physiologic Ossification)) OR (Ossification, Physiological)) OR (Physiological Ossification)) OR (Ossification, Physiologic))) | 91 |
