# Supplementary material for: The impact of Zn-doped synthetic polymer materials on bone regeneration: a systematic review
Source: Stem Cell Res Ther. 2021 Feb 12;12:123. doi: 10.1186/s13287-021-02195-y (PMC7881550; doi:10.1186/s13287-021-02195-y)
Supplement: Supplementary file 2 — Additional file 2 : Table S2. Search strategies in Embase database and related results. [file 13287_2021_2195_MOESM2_ESM.docx]

**Table S2.** Search strategies in Embase database and related results.

| **Search** | **Search Strategies** | **Number of Publications** |
| --- | --- | --- |
| #1 | 'zinc'/exp | 116,567 |
| #2 | zn | 90,363 |
| #3 | #1 OR #2 | 158,437 |
| #4 | 'polymer'/exp | 706,962 |
| #5 | polymers | 100,075 |
| #6 | synthetic AND polymers | 9,292 |
| #7 | polyester | 15,800 |
| #8 | pla | 67,027 |
| #9 | polylactic AND acid | 11,592 |
| #10 | pga | 8,407 |
| #11 | polyglycolic AND acid | 6,438 |
| #12 | plga | 13,194 |
| #13 | poly AND 'lactic co glycolic' AND acid | 5,820 |
| #14 | pcl | 16,846 |
| #15 | polycaprolactone | 10,747 |
| #16 | pu | 49,913 |
| #17 | polyurethane | 13,171 |
| #18 | peg | 62,394 |
| #19 | polyethylene AND glycol | 33,862 |
| #20 | pbt | 2,326 |
| #21 | polybutylene AND terephthalate | 272 |
| #22 | paa | 6,351 |
| #23 | polyacrylic AND acid | 6,529 |
| #24 | peo | 4,907 |
| #25 | polyethylene AND oxide | 5,302 |
| #26 | pva | 10,055 |
| #27 | polyvinyl AND alcohol | 15,454 |
| #28 | pdo | 1,399 |
| #29 | polydioxanone | 2,687 |
| #30 | #4 OR #5 OR #6 OR #7 OR #8 OR #9 OR #10 OR #11 OR #12 OR #13 OR #14 OR #15 OR #16 OR #17 OR #18 OR #19 OR #20 OR #21 OR #22 OR #23 OR #24 OR #25 OR #26 OR #27 OR #28 OR #29 | 916,541 |
| #31 | 'ossification'/exp | 48,914 |
| #32 | osteogenesis | 30,767 |
| #33 | bone AND formation | 115,406 |
| #34 | ossifications | 1,372 |
| #35 | osteoclastogenesis | 13,334 |
| #36 | osteoclastogeneses | 0 |
| #37 | endochondral AND ossification | 5,348 |
| #38 | endochondral AND ossifications | 29 |
| #39 | ossification, AND endochondral | 5,348 |
| #40 | ossifications, AND endochondral | 29 |
| #41 | physiologic AND ossification | 533 |
| #42 | ossification, AND physiological | 2,130 |
| #43 | physiological AND ossification | 2,130 |
| #44 | ossification, AND physiologic | 533 |
| #45 | #31 OR #32 OR #33 OR #34 OR #35 OR #36 OR #37 OR #38 OR #39 OR #40 OR #41 OR #42 OR #43 OR #44 | 163,095 |
| #46 | #3 AND #30 AND #45 | 59 |
