# Supplementary material for: The impact of Zn-doped synthetic polymer materials on bone regeneration: a systematic review
Source: Stem Cell Res Ther. 2021 Feb 12;12:123. doi: 10.1186/s13287-021-02195-y (PMC7881550; doi:10.1186/s13287-021-02195-y)
Supplement: Supplementary file 3 — Additional file 3 : Table S3. Search strategies in Cochrane Library database and related results. [file 13287_2021_2195_MOESM3_ESM.docx]

**Table S3.** Search strategies in Cochrane Library database and related results.

| **Search** | **Search Strategies** | **Number of Publications** |
| --- | --- | --- |
| #1 | MeSH descriptor: [Zinc] explode all trees | 1619 |
| #2 | Zn | 1326 |
| #3 | #1 OR #2 | 2575 |
| #4 | MeSH descriptor: [Polymers] explode all trees | 18647 |
| #5 | Polymer | 2163 |
| #6 | synthetic polymers | 85 |
| #7 | polyester | 426 |
| #8 | PLA | 2947 |
| #9 | polylactic acid | 215 |
| #10 | PGA | 1630 |
| #11 | Polyglycolic acid | 305 |
| #12 | PLGA | 53 |
| #13 | poly(lactic-co-glycolic acid) | 20 |
| #14 | PCL | 437 |
| #15 | Polycaprolactone | 19 |
| #16 | PU | 2406 |
| #17 | polyurethane | 636 |
| #18 | PEG | 5086 |
| #19 | Polyethylene glycol | 2124 |
| #20 | PBT | 153 |
| #21 | polybutylene terephthalate | 2 |
| #22 | PAA | 1328 |
| #23 | Polyacrylic acid | 80 |
| #24 | PEO | 31 |
| #25 | polyethylene oxide | 137 |
| #26 | PVA | 173 |
| #27 | polyvinyl alcohol | 275 |
| #28 | PDO | 28 |
| #29 | polydioxanone | 171 |
| #30 | #4 OR #5 OR #6 OR #7 OR #8 OR #9 OR #10 OR #11 OR #12 OR #13 OR #14 OR #15 OR #16 OR #17 OR #18 OR #19 OR #20 OR #21 OR #22 OR #23 OR #24 OR #25 OR #26 OR #27 OR #28 OR #29 | 35074 |
| #31 | MeSH descriptor: [Osteogenesis] explode all trees | 335 |
| #32 | Bone Formation | 3559 |
| #33 | Ossification | 1198 |
| #34 | Ossifications | 57 |
| #35 | Osteoclastogenesis | 73 |
| #36 | Osteoclastogeneses | 0 |
| #37 | Endochondral Ossification | 5 |
| #38 | Endochondral Ossifications | 0 |
| #39 | Ossification, Endochondral | 5 |
| #40 | Ossifications, Endochondral | 0 |
| #41 | Physiologic Ossification | 7 |
| #42 | Ossification, Physiological | 27 |
| #43 | Physiological Ossification | 27 |
| #44 | Ossification, Physiologic | 7 |
| #45 | #31 OR #32 OR #33 OR #34 OR #35 OR #36 OR #37 OR #38 OR #39 OR #40 OR #41 OR #42 OR #43 OR #44 | 4250 |
| #46 | #3 AND #30 AND #45 | 3 |
